# Entrectinib in *ROS1*-positive advanced non-small cell lung cancer: the phase 2/3 BFAST trial

**DOI:** 10.1038/s41591-024-03008-4

**Published:** 2024-06-19

**Authors:** Solange Peters, Shirish M. Gadgeel, Tony Mok, Ernest Nadal, Saadettin Kilickap, Aurélie Swalduz, Jacques Cadranel, Shunichi Sugawara, Chao-Hua Chiu, Chong-Jen Yu, Mor Moskovitz, Tomohiro Tanaka, Rhea Nersesian, Sarah M. Shagan, Margaret Maclennan, Michael Mathisen, Vijay Bhagawati-Prasad, Cheick Diarra, Zoe June Assaf, Venice Archer, Rafal Dziadziuszko

**Affiliations:** 1 Lausanne University Hospital, Centre Hospitalier Universitaire Vaudois (CHUV), Lausanne, Switzerland; 2 Henry Ford Cancer Institute/Henry Ford Health System, Detroit, MI USA; 3 State Laboratory of Translational Oncology, The Chinese University of Hong Kong, Prince of Wales Hospital, Hong Kong, Hong Kong SAR; 4 Thoracic Oncology Unit, Department of Medical Oncology, Catalan Institute of Oncology (ICO), L’Hospitalet de Llobregat, Barcelona, Spain; 5 Department of Medical Oncology, Instinye University Faculty of Medicine, Istanbul, Turkey; 6 Department of Medical Oncology, Centre Léon Bérard, Lyon, France; 7 Department of Pneumology and Thoracic Oncology, APHP, Hôpital Tenon and GRC04 Theranoscan Sorbonne Université, Paris, France; 8 Department of Pulmonary Medicine, Sendai Kousei Hospital, Sendai, Japan; 9 Department of Chest Medicine, Taipei Veterans General Hospital, Taipei, Taiwan; 10 Taipei Cancer Center and Taipei Medical University Hospital, Taipei Medical University, Taipei, Taiwan; 11 Department of Internal Medicine, National Taiwan University Hospital Hsin-Chu Branch, Hsinchu, Taiwan; 12 Thoracic Cancer Service, Davidoff Cancer Center, Rabin Medical Center, Beilinson Campus, Petah Tikva, Israel; 13 Chugai Pharmaceutical Co. Ltd, Tokyo, Japan; 14 Genentech Inc., South San Francisco, CA USA; 15 Syneos Health, Edinburgh, UK; 16 F. Hoffmann-La Roche Ltd, Welwyn Garden City, UK; 17 Department of Oncology and Radiotherapy and Early Clinical Trials Unit, Medical University of Gdansk, Gdansk, Poland

**Keywords:** Cancer, Biomarkers

## Abstract

Although comprehensive biomarker testing is recommended for all patients with advanced/metastatic non-small cell lung cancer (NSCLC) before initiation of first-line treatment, tissue availability can limit testing. Genomic testing in liquid biopsies can be utilized to overcome the inherent limitations of tissue sampling and identify the most appropriate biomarker-informed treatment option for patients. The Blood First Assay Screening Trial is a global, open-label, multicohort trial that evaluates the efficacy and safety of multiple therapies in patients with advanced/metastatic NSCLC and targetable alterations identified by liquid biopsy. We present data from Cohort D (*ROS1*-positive). Patients ≥18 years of age with stage IIIB/IV, *ROS1*-positive NSCLC detected by liquid biopsies received entrectinib 600 mg daily. At data cutoff (November 2021), 55 patients were enrolled and 54 had measurable disease. Cohort D met its primary endpoint: the confirmed objective response rate (ORR) by investigator was 81.5%, which was consistent with the ORR from the integrated analysis of entrectinib (investigator-assessed ORR, 73.4%; data cutoff May 2019, ≥12 months of follow-up). The safety profile of entrectinib was consistent with previous reports. These results demonstrate consistency with those from the integrated analysis of entrectinib in patients with *ROS1*-positive NSCLC identified by tissue-based testing, and support the clinical value of liquid biopsies to inform clinical decision-making. The integration of liquid biopsies into clinical practice provides patients with a less invasive diagnostic method than tissue-based testing and has faster turnaround times that may expedite the reaching of clinical decisions in the advanced/metastatic NSCLC setting. ClinicalTrials.gov registration: NCT03178552.

## Abstract (web summary)

Results from this single-arm cohort of the BFAST trial showed that the clinical efficacy of entrectinib in patients with *ROS1*-positive NSCLC, selected using liquid biopsies, is consistent with that seen in previous reports where patients were selected using tissue-based testing methods.

## Main

The development of highly effective targeted therapies has improved survival outcomes for patients with oncogene-driven, advanced NSCLC, and targeted agents are now standard of care for these patients^1^. As such, comprehensive biomarker testing to identify the presence of oncogenic driver alterations (including various *EGFR* mutations*,* anaplastic lymphoma kinase (*ALK*), *RET*, *NTRK* 1/2/3, *ROS1*, *BRAF* V600E, *MET*ex14 skipping and *ERBB2*) is recommended for patients with advanced/metastatic NSCLC before initiation of first-line treatment, except for patients with *KRAS* G12C mutation, *ERBB2* mutation or *EGFR* exon 20 insertion mutation, where targeted therapy is recommended as a second-line treatment^2,3^. Despite these recommendations, a recent report of real-world data from the US Oncology Network suggests that the percentage of patients with metastatic NSCLC who receive molecular testing for multiple targetable biomarkers via next-generation sequencing (NGS) remains low^4^. Another report, assessing the clinical practice gaps on the implementation of personalized medicine in advanced NSCLC, found that approximately 50% of patients do not receive targeted therapies due to factors associated with obtaining biomarker test results^5^.

One factor that can limit molecular testing in patients with advanced NSCLC is tissue availability. Tissue biopsies may not always be feasible, due to either the patient’s comorbidities or the location of their tumor^1^. Alternatively, the yield of viable tumor cells collected during biopsy may be too low for molecular testing^6^. Furthermore, repeat biopsies are associated with risk of complications and are undesirable from the patient’s perspective^7^. Genomic testing in liquid biopsies can be utilized to overcome the inherent limitations of tissue sampling^6^. These liquid biopsies can detect molecular alterations in either circulating tumor DNA (ctDNA) or, less commonly, circulating tumor cells^8^, and are recommended for identification of patients with oncogene-driven NSCLC that can be therapeutically targeted^3,9^. Studies have demonstrated high concordance between tissue and liquid biopsies, albeit that the latter are less sensitive than the former^10,11^. Despite this, due to their faster turnaround time compared with tissue-based testing and equivalent time-to-treatment, liquid biopsies are commonly used as a first-line diagnostic and have demonstrated clinical benefit^12,13^. In addition to the identification of genomic alterations, liquid biopsies can also be used to explore mechanisms of resistance to kinase inhibitors^14^. For patients with adequate tissue sample available, liquid biopsies can be used in parallel with tissue-based assays, immunohistochemistry or fluorescence in situ hybridization to facilitate more extensive testing^3,6^.

The ROS proto-oncogene 1 (*ROS1*) gene encodes for the ROS1 tyrosine kinase, and rearrangements in *ROS1* can result in constitutively active fusion oncoproteins^15,16^. *ROS1* fusions occur in a variety of different tumor types, including in 1–2% of NSCLC cases^15,17,18^. Brain metastases are common in patients with *ROS1-*positive, advanced NSCLC, having been detected in approximately 40% of cases^19^, which highlights the need for central nervous system (CNS)-penetrating treatments with proven intracranial efficacy for these patients^20,21^. Entrectinib is a potent ROS1, TRK and ALK tyrosine kinase inhibitor (TKI) that was specifically developed for its ability to cross the blood–brain barrier and remain within the CNS^22,23^. Results from an integrated analysis of three phase 1/2 studies, ALKA-372-001 (EudraCT 2012-000148-88), STARTRK-1 (NCT02097810) and STARTRK-2 (NCT02568267), have demonstrated deep and durable responses with entrectinib in patients with *ROS1*-positive NSCLC^23–26^. In the efficacy-evaluable population (*n* = 172; data cutoff 2 August 2021), ORR was 67% (95% confidence interval (CI): 59.9–74.4) with a median duration of response (DoR) of 20.4 months (95% CI: 14.8–34.8) and median progression-free survival (PFS) of 16.8 months (95% CI: 12.2–22.4)^23^. Entrectinib also yielded durable intracranial responses in patients with baseline CNS metastases by blinded independent central review (*n* = 51; intracranial ORR 49%, median intracranial DoR 12.9 months)^23^. These trials enrolled patients identified as having *ROS1*-positive NSCLC using traditional tissue-based testing.

**Fig. 1:**
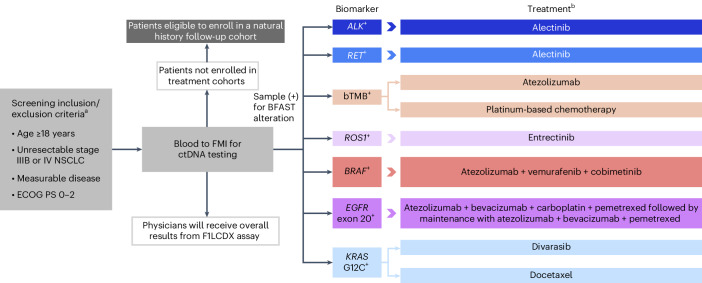
BFAST study design. ^a^All cohorts have additional, treatment-specific inclusion/exclusion criteria. ^b^Please see ClinicalTrials.gov for full treatment dosing information. Figure adapted with permission from Peled, N. et al. Higher dose alectinib for advanced *RET*+ NSCLC: results from the *RET*+ cohort of the Blood First Assay Screening Trial (BFAST), presented at the 2020 World Conference on Lung Cancer (28–31 January 2021, Singapore). bTMB, blood tumor mutational burden; FMI, Foundation Medicine, Inc.; IV, intravenous.

The Blood First Assay Screening Trial (BFAST; NCT03178552; Fig. 1) is a global, open-label, multicohort trial evaluating the efficacy and safety of targeted therapies or immunotherapy in patients with advanced/metastatic NSCLC harboring actionable genetic alterations detected solely by genomic testing in liquid biopsies. Data from the *ALK*-positive cohort (Cohort A) and the tumor mutational burden (TMB)-high cohort (Cohort C) of BFAST have been published previously^13,27^. Data from Cohort A demonstrated the clinical application of liquid biopsies in identification of patients with *ALK*-positive NSCLC to be treated with alectinib^13^. Cohort C did not meet its primary endpoint of investigator-assessed PFS in patients with blood TMB of ≥16 (ref. ^27^). Although Cohort C did not meet its primary endpoint, previous studies have demonstrated that TMB status already identified by liquid biopsy can predict response to cancer immunotherapy^28,29^, suggesting that exploration of additional cutfoffs for this biomarker may be warranted.

We present efficacy and safety data from Cohort D of BFAST, an evaluation of entrectinib in treatment-naive patients with *ROS1*-positive NSCLC identified using NGS testing in liquid biopsies alone. The objective of this study is to demonstrate consistency in data between when patients with *ROS1*-positive NSCLC are identified via liquid biopsies and when they are identified via tissue-based testing (integrated analysis of entrectinib; data cutoff 1 May 2019).

## Results

### Patients

Between 11 January 2018 and 9 December 2020, 5,220 patients were screened of whom 92 were identified to have *ROS1*-positive, advanced/metastatic NSCLC by liquid biopsies, giving a prevalence of *ROS1* fusions of 1.8%. Of these 92 patients, 55 treatment-naive patients were enrolled of whom 54 had measurable disease (Table 1). The median age was 56 years (range 22–83); 58% (*n* = 32) of patients were female and 75% (*n* = 41) had no history of tobacco use. Nonsquamous adenocarcinoma was the most common histology (*n* = 48, 94%), and four patients (7.3%) had asymptomatic and/or previously treated investigator-assessed CNS metastases at baseline. The median duration of follow-up was 18.3 months; the last patient included in this analysis was enrolled on 1 October 2020 and the data cutoff was 26 November 2021. At the time of primary analysis, 32 patients (58%) remained in the study of whom 15 (27%) were still receiving study treatment. Forty patients (73%) discontinued study treatment, for reasons including progressive disease (PD; *n* = 28, 51%), adverse events (AEs) (*n* = 4, 7.3%), consent withdrawal by subject (*n* = 4, 7.3%), death (*n* = 3, 5.5%) and symptomatic deterioration (*n* = 1, 1.8%).

**Table 1:** Demographics, baseline characteristics and disease history

	*ROS1*-positive NSCLC (*n* = 55)^a^
Median age, years (range)	56 (22–83)
Sex, *n* (%)	
Female/male	32 (58)/23 (42)
Ethnicity, *n* (%)	
Asian/white/other/unknown	16 (29)/26 (47)/2 (4)/11 (20)
Tobacco use history, *n* (%)	
Yes (previous or current)/no	14 (25)/41 (75)
ECOG PS, *n* (%)	
0/1/2	25 (45)/29 (53)/1 (2)
CNS metastases, *n* (%)	
Present/absent by INV	4 (7)/51 (93)
Present/absent by IRF	1 (2)/54 (98)
Histology (nonsquamous), *n* (%)	*n* = 51
Adenocarcinoma/NSCLC or NOS/sarcomatoid	48 (94)/2 (4)/1 (2)
Histology (squamous), *n* (%)	*n* = 4
Adenosquamous^b^/squamous	2 (50) / 2 (50)
Staging at initial diagnosis, *n* (%)	
IIIA/IIIB/IV	7 (13)/3 (5)/45 (82)
*ROS1* fusion partner, *n* (%)	
* CD74* fusion or rearrangement	31 (56.4)
* EZR* fusion	13 (23.6)
* TPM3* fusion	4 (7.3)
* ROS1* self-rearrangement	2 (3.6)
* FAM91A1* rearrangement	1 (1.8)
* LRIG3* fusion	1 (1.8)
* RFC4* rearrangement	1 (1.8)
* SDC4* fusion	1 (1.8)
* ZCCHC8* fusion	1 (1.8)

BFAST Cohort D data cutoff, 26 November 2021. NOS, not otherwise specified.

^a^*n* = 54 with measurable disease.

^b^Predominantly squamous.

### Efficacy

The median duration of follow-up in Cohort D was 18.3 months. Responses were assessed in patients with measurable disease at baseline (*n* = 54). Forty-four patients had a response (confirmed ORR of 81.5%; 95% CI: 68.6–90.8) by both investigator (INV; primary endpoint) and independent review facility (IRF) assessment (Table 2). Two patients had a complete response (CR) and 42 a partial response (PR) by investigator assessment; three patients had a CR and 41 a PR by IRF assessment. In the tissue-based integrated analysis of entrectinib in *ROS1*-positive NSCLC, at the 1 May 2019 cutoff 94 patients with ≥12 months of follow-up were enrolled and the median duration of follow-up was comparable to that from BFAST Cohort D (20.9 versus 18.3 months, respectively). Because the investigator-assessed confirmed ORR was higher than the protocol-defined threshold of 70.4% (95% CI: 56.0–82.0), Cohort D met its primary endpoint demonstrating a consistent ORR with that from the integrated analysis of entrectinib (investigator-assessed ORR, 73.4% (95% CI: 63.3–82.0)).

**Table 2:** Overall efficacy of entrectinib in patients with *ROS1*-positive NSCLC

Efficacy parameter	*ROS1*-positive NSCLC (*n* = 54)	
	INV assessment	IRF assessment
ORR, *n* (%)	44 (81.5)	44 (81.5)
95% CI	68.6–90.8	68.6–90.8
CR, *n* (%)	2 (3.7)	3 (5.6)
PR, *n* (%)	42 (77.8)	41 (75.9)
SD, *n* (%)	7 (13.0)	7 (13.0)
PD, *n* (%)	3 (5.6)	1 (1.9)
Missing/nonevaluable (NE)	0	2 (3.7)
CBR^a^, *n* (%) 95% CI	47 (87.0) 75.1–94.6	44 (81.5) 68.6–90.8
Median DoR, months (95% CI)	*n* = 44 13.0 (6.3–18.4)	*n* = 44 16.7 (5.6–24.0)
Responders with event, *n* (%)	30 (68.2)	25 (56.8)
12-month event-free rate, %	53.2	57.3
Median time to CNS progression, months (95% CI)	*n* = 54 NE (NE)	*n* = 54 NE (NE)
Patients with event, *n* (%)	9 (16.7)	6 (11.1)
12-month event-free rate, %	83.5	86.4
Median PFS, months (95% CI)	*n* = 55 12.9 (8.7–18.5)	*n* = 55 14.8 (7.2–24.0)
Patients with event, *n* (%)	39 (70.9)	33 (60.0)
12-month event-free rate, %	50.7	52.4
OS	*n* = 55	
Patients with event, *n* (%)	20 (36.4)	
12-month event-free rate, %	79.0	

BFAST Cohort D data cutoff date, 26 November 2021.

^a^CBR, CR + PR + SD ≥ 24 weeks.

Clinical benefit rate (CBR) was 87.0% (*n* = 47; 95% CI: 75.1–94.6) by investigator and 81.5% (*n* = 44; 95% CI: 75.1–94.6) by IRF assessment (Table 2). In the four patients with invesigator-assessed CNS metastases at baseline, two had PR.

**Fig. 2:**
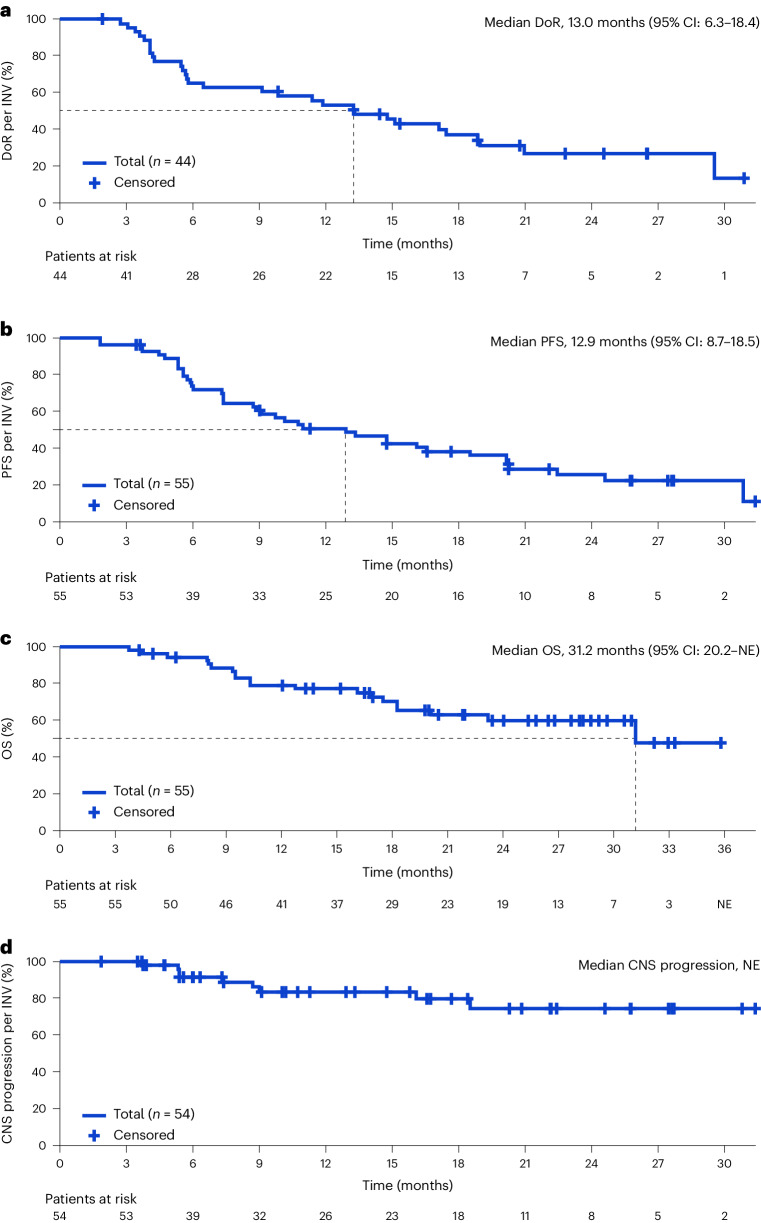
Clinical outcomes of patients with *ROS1*-positive NSCLC who were identified by liquid biopsy and treated with entrectinib. **a**–**d**, DoR (*n* = 44) (**a**), PFS (*n* = 55) (**b**), OS (*n* = 55) (**c**) and CNS progression (*n* = 54) (**d**) Kaplan–Meier curves for patients with *ROS1*-positive NSCLC who were identified via liquid biopsies and treated with entrectinib. BFAST Cohort D data cutoff, 26 November 2021. DoR, PFS and CNS progression were assessed by investigator. NE, not estimable; INV, investigator.

Among responders (*n* = 44) the median DoR was 13.0 months (95% CI: 6.3–18.4) by investigator and 16.7 months (95% CI: 5.6–24.0) by IRF (Table 2 and Fig. 2a). Median PFS (*n* = 55) was 12.9 months (95% CI: 8.7–18.5) by investigator and 14.8 months (95% CI: 7.2–24.0) by IRF (Table 2 and Fig. 2b). Overall survival (OS; *n* = 55) data were immature, with 20 events (36.4%) recorded (Table 2 and Fig. 2c); 12-month OS probability was 79.0%. Median time to CNS progression (*n* = 54) was not reached (Table 2 and Fig. 2d), and 12-month CNS progression-free rate was 83.5% by investigator and 86.4% by IRF.

### Safety

All 55 patients enrolled in Cohort D received one or more doses of entrectinib and were included in the safety population. The median duration of entrectinib treatment was 12.8 months (range 1–33). Most treatment-related AEs (TRAEs) were nonserious and there were no treatment-related deaths (Table 3). Seven patients (12.7%) experienced one or more of the following serious TRAEs: cerebellar syndrome, cognitive disorder, memory impairment, cardiac failure, left ventricular dysfunction, interstitial lung disease, pleural effusion, ankle fracture and fluid retention (each *n* = 1). Grade 3–5 AEs were reported in 56.4% (*n* = 31) of patients; of these, weight gain was the most common (*n* = 4, 7.3%; all grade 3; Table 3). Two grade 5 AEs were reported on the study: one was due to COVID-19 and the other was unexplained and deemed not related to study treatment by the investigator. TRAEs led to dose interruption, reduction or discontinuation in 20.0% (*n* = 11), 36.4% (*n* = 20) and 5.5% (*n* = 3) of patients, respectively. The median dose intensity of entrectinib was 97.5% (range 31.8–103.2) and the median number of doses received was 362 (range 32–1,000).

**Table 3:** Safety summary

	*ROS1*-positive NSCLC (*n* = 55)
Patients with ≥1 TRAE, *n* (%)	51 (92.7)
Patients with ≥1 serious TRAE, *n* (%)	7 (12.7)
Patients with TRAE leading to, *n* (%)	
Dose reduction	20 (36.4)
Dose interruption	11 (20)
Dose discontinuation	3 (5.5)
Patients with AE leading to death^a^, *n* (%)	2 (3.6)
Patients with grade 3–5 AE, *n* (%)	31 (56.4)
Patients with grade 3–5 AE with incidence of ≥2%, *n* (%)	
Weight increase	4 (7.3)
Syncope	3 (5.5)
Pleural effusion	3 (5.5)
Dizziness	2 (3.6)
Cardiac failure	2 (3.6)
Pulmonary embolism	2 (3.6)
Urinary tract infection	2 (3.6)

BFAST Cohort D data cutoff, 26 November 2021. ^a^Grade 5 AEs: COVID-19 and unexplained AE deemed not related to study treatment by investigator (both *n* = 1).

### Biomarker analyses

Patients were screened prospectively for actionable alterations using either the Foundation Medicine blood-based NGS assay, FoundationOneLiquid CDx clinical trial assay (*n* = 22) or its predecessor, Foundation Medicine Assay for Circulating Tumor DNA (FoundationACT (*n* = 33)). High concordance was demonstrated between assays used to identify *ROS1* fusions (96.9% positive predictive agreement between FoundationOneLiquid CDx clinical trial assay and FoundationACT, as described in Supplementary Data).

**Fig. 3:**
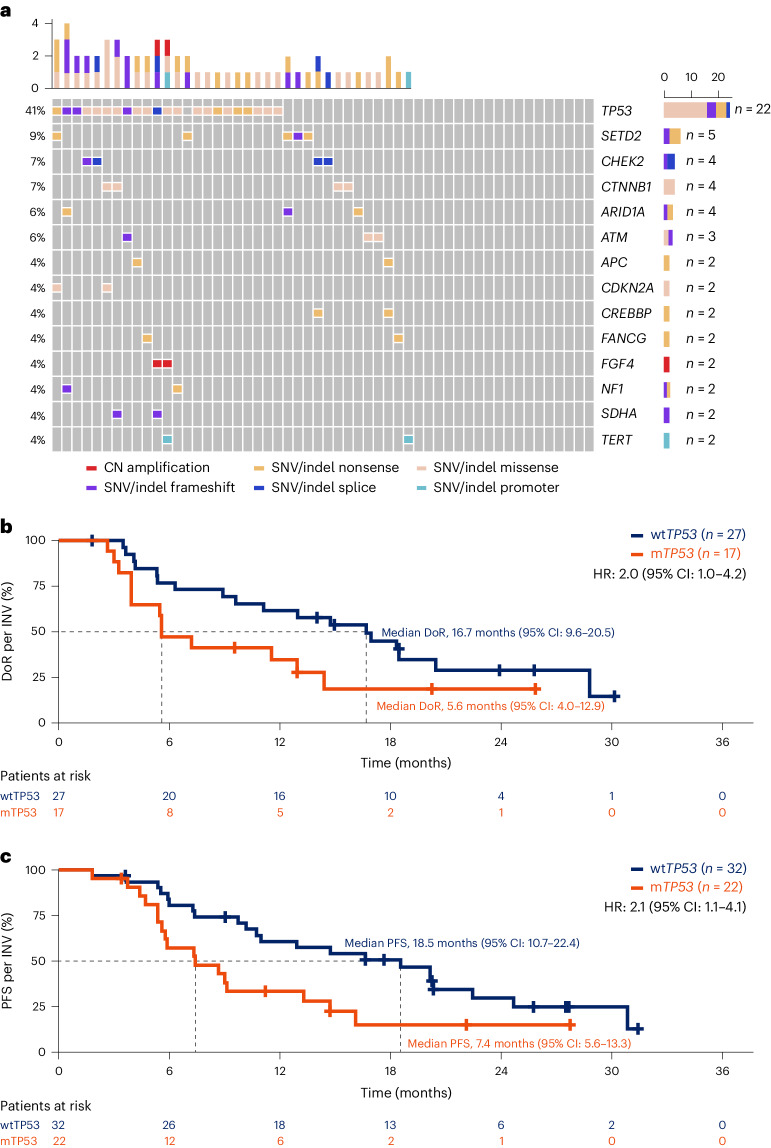
Comutations identified in patients with *ROS1*-positive NSCLC at baseline and clinical outcomes of patients with m*TP53* versus wt*TP53.* **a**, Comutations and type of mutation identified in patients with *ROS1*-positive NSCLC via liquid biopsy at baseline; the most common comutation identified in patients from BFAST Cohort D was *TP53*. **b**,**c**, DoR (*n* = 44) (**b**) and PFS (*n* = 54) (**c**) Kaplan–Meier curves for patients from BFAST Cohort D with m*TP53* (red) versus wt*TP53* (blue). Patients with m*TP53* had numerically shorter DoR and PFS compared with those with wt*TP53* at baseline. CN, copy number.

**Extended Data Fig. 1:**
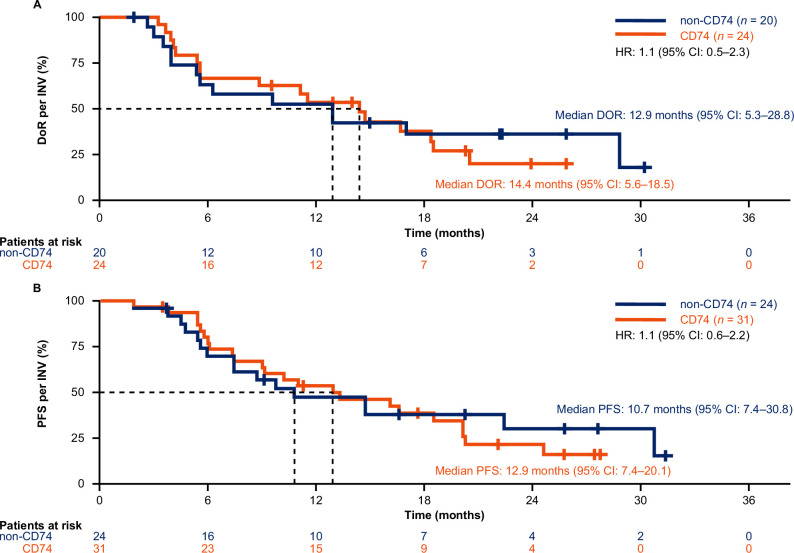
Clinical outcomes of patients treated with entrectinib and who had *CD74* as the *ROS1* fusion partner and those with other (non-*CD74*) *ROS1* fusion partners. (**A**) DoR (n = 44) and (**B**) PFS (n = 55) Kaplan–Meier curves for patients who had CD74 as the ROS1 fusion partner (red) and those with other (non-*CD74*) *ROS1* fusion partners (blue). There was no difference in median DoR and PFS between patients who had *CD74* as the *ROS1* fusion partner (n = 31) and those with other fusion partners (n = 24). One patient who had *CD74* as the *ROS1* fusion partner had non-measurable disease. CI, confidence intervals; DoR, duration of response; HR, hazard ratio; INV, investigator; PFS, progression-free survival; *ROS1*, ROS proto-oncogene 1.

**Extended Data Fig. 2:**
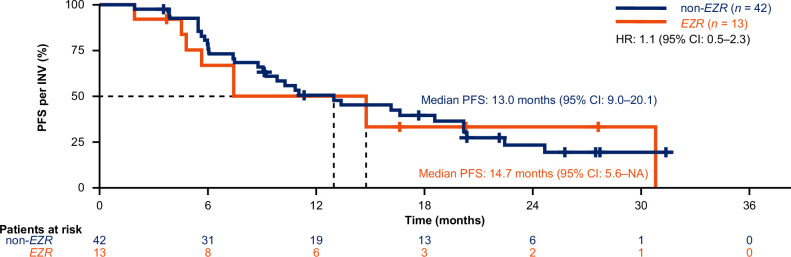
PFS in patients treated with entrectinib who had *EZR* as the *ROS1* fusion partner and those with other (non-*EZR*) *ROS1* fusion partners. Median PFS was similar between patients who had *EZR* as the *ROS1* fusion partner (n = 13) and those with other fusion partners (n = 42). CI, confidence intervals; HR, hazard ratio; INV, investigator; NA, not available; PFS, progression-free survival; *ROS1*, ROS proto-oncogene.

**Extended Data Fig. 3:**
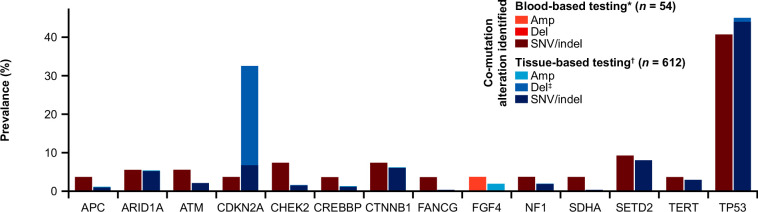
Prevalence of *ROS1* fusion co-mutations identified by liquid biopsy and tissue-based testing. The prevalence of comutations that were identified by liquid biopsies in BFAST Cohort D were comparable with those identified by tissue-based testing from the FMCore database. A low prevalence of rearrangements were identified in the tissue-based testing data; these were identified in the following genes: *CDKN2A* (n = 8), *NF1* (n = 3), *APC* (n = 1), *ARID1A* (n = 1), *CREBBP* (n = 1), *CTNNB1* (n = 1), and *TP53* (n = 1). *Patients who had liquid biopsies were enrolled in BFAST Cohort D (n = 54) and testing was conducted using FoundationOne®Liquid CDx clinical trial assay or FoundationACT™ (Foundation Medicine, Inc., Cambridge, MA). ^†^Patients with NSCLC from the FMCore database (N = 612) who were *ROS1*-positive by tissue-based NGS testing with FoundationOne®CDx (Data cut-off: April 2023)30. ^‡^Copy number deletions are not accurately detected in liquid biopsies due to limited sensitivity, which may in part explain the high prevalence of *CDKN2A* deletions identified in the tissue-based assay. Amp, copy number amplification; Del, copy number deletion; FoundationACT™, Foundation Medicine Assay for Circulating Tumor DNA; NGS, next generation sequencing; NSCLC, non-small cell lung cancer; *ROS1*, ROS proto-oncogene 1; SNV/indel, short variant.

Nine different *ROS1* fusion partners were identified (Table 1), the most common being *CD74* (*n* = 31, 56.4%), *EZR* (*n* = 13, 23.6%), *TPM3* (*n* = 4, 7.3%) and *ROS1* self-rearrangement (*n* = 2, 3.6%). There was no difference in best overall response between patients who had *CD74* as the *ROS1* fusion partner (*n* = 30) and those with other fusion partners (*n* = 24; Supplementary Table 1). Similarly, there was no difference in DoR and PFS between patients who had *CD74* as the *ROS1* fusion partner (*n* = 31) and those with other fusion partners (*n* = 24; Extended Data Fig. 1). The second-most common *ROS1* fusion partner identified in patients was *EZR*; PFS was similar between patients who had *EZR* as the *ROS1* fusion partner (*n* = 13) and those with other fusion partners (*n* = 42; Extended Data Fig. 2). Comutations reported at baseline are shown in Fig. 3 (*n* = 54); the prevalence of comutations identified by liquid biopsies in BFAST Cohort D was comparable to that identified by tissue-based testing from the FMCore database (patients with NSCLC in the FMCore database with *ROS1* rearrangement, *n* = 612; Extended Data Fig. 3)^30^. The most common comutation identified in patients from BFAST Cohort D was *TP53* (*n* = 22, 40.7%; Fig. 3a). Patients with mutant *TP53* (m*TP53)* had numerically shorter DoR and PFS compared with those with wild-type *TP53* (wt*TP53*) at baseline (Fig. 3b,c).

**Extended Data Fig. 4:**
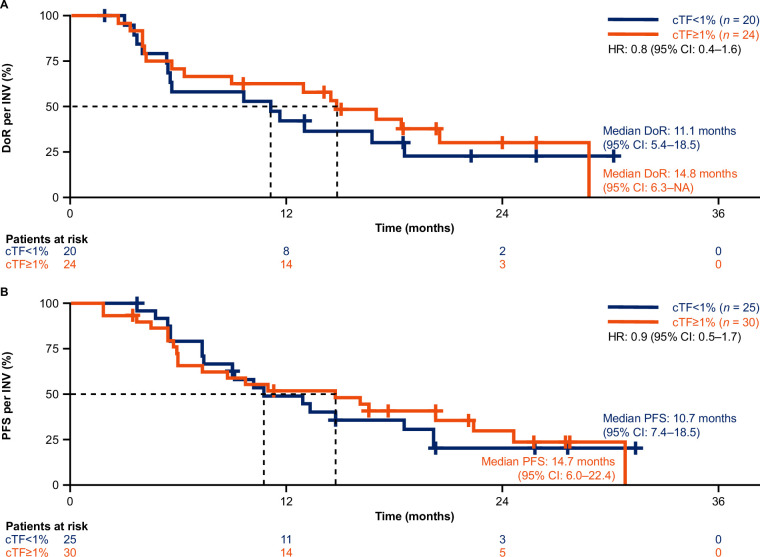
Clinical outcomes of patients treated with entrectinib and had low ( < 1%) and those with high ( ≥ 1%) baseline ctDNA fraction. (A) DoR (n = 44) and (B) PFS (n = 55) Kaplan–Meier curves for patients who had low ( < 1%, blue) and those with high ( ≥ 1%, red) baseline ctDNA fraction. There was no difference in median DoR or median PFS between patients with a baseline cTF <1% and those with a baseline cTF ≥1%. CI, confidence intervals; cTF, ctDNA fraction; DoR, duration of response; HR, hazard ratio; INV, investigator; NA, not available; PFS, progression-free survival.

**Extended Data Fig. 5:**
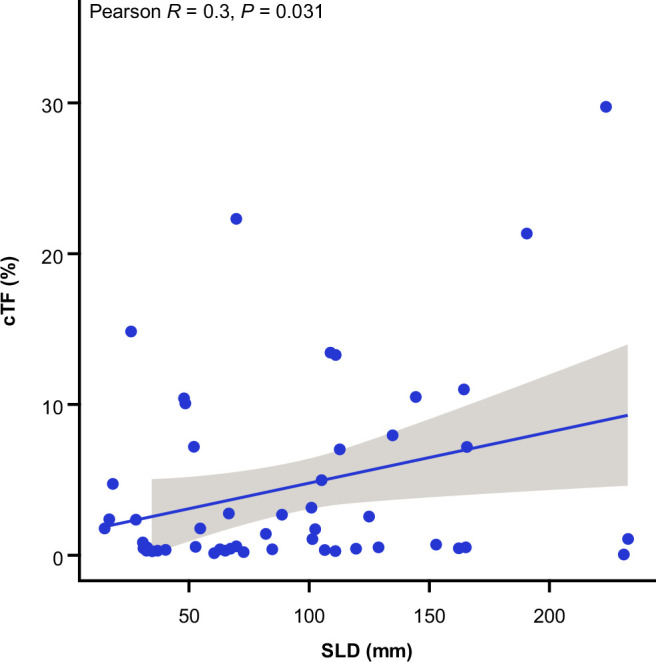
Correlation of baseline cTF with tumor size as measured by SLD. cTF was derived from patients who have been screened via FoundationACT™ or FoundationOne® Liquid CDx assays and individual values have been plotted (n = 52*). A linear regression was conducted using the stat_smooth() R function with method set to “lm” and 95% CI bands are shown. Pearson’s correlation coefficient testing (two-sided) identified a weak but positive association between baseline cTF and tumor burden. *Three patients had missing SLD. cTF, ctDNA fraction; FoundationACT™, Foundation Medicine Assay for Circulating Tumor DNA; SLD, sum of the longest diameters.

A post hoc exploratory analysis was carried out to determine whether the amount of ctDNA in the blood, as measured by estimated circulating tumor fraction (cTF) at baseline, was associated with clinical outcomes. There was no difference in either median DoR or median PFS between patients with baseline cTF <1% and those with baseline cTF ≥1% (Extended Data Fig. 4). Additional cTF thresholds were also assessed, with no difference found in outcomes between groups (Supplementary Fig. 1). Further analyses showed a weak but positive association between baseline cTF and tumor burden (evaluated by the sum of the longest diameters (SLD); Extended Data Fig. 5).

**Extended Data Fig. 6:**
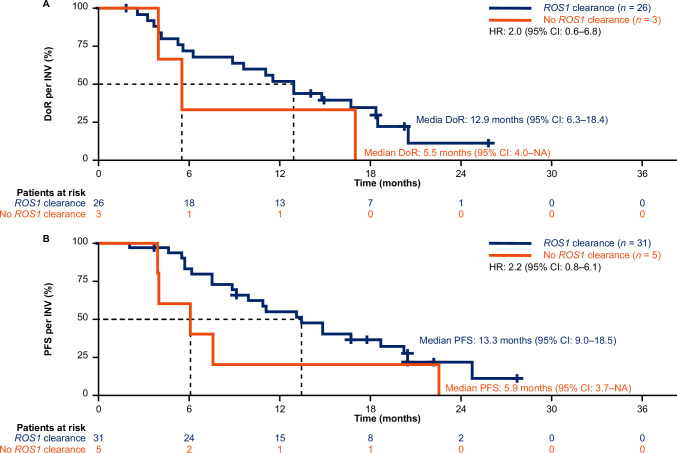
Clinical outcomes of patients treated with entrectinib and cleared *ROS1* from the ctDNA by C3D1 and those who did not. (A) DoR (n = 29) and (B) PFS (n = 36) Kaplan–Meier curves for patients who cleared ROS1 from the ctDNA by C3D1 (blue) and those who did not (red). *ROS1* clearance was associated with longer median DoR and median PFS compared with lack of clearance. 36 patients had plasma samples from C3D1, one of these patients had non-measurable disease. CI, confidence interval; ctDNA, circulating tumor DNA; DoR, duration of response; HR, hazard ratio; INV, investigator; NA, not available; PFS, progression-free survival; *ROS1*, ROS proto-oncogene 1.

ctDNA clearance was evaluated in a subset of 36 patients who had plasma samples from cycle 3, day 1 (C3D1). Most patients (*n* = 31, 86.1%) had cleared ctDNA as assessed by the absence of *ROS1* from baseline to C3D1. Twenty-six patients (89.7%) who responded to treatment with entrectinib (and had plasma samples available) had cleared *ROS1* by C3D1 (Supplementary Table 2). *ROS1* clearance was also associated with longer median DoR and median PFS compared with lack of clearance (Extended Data Fig. 6). Of the five patients who did not clear *ROS1* by C3D1, three had PR and two had stable disease (SD) as their confirmed best overall response. The confirmed DoRs of the three patients who achieved PR were 4.0, 5.5 and 17.0 months. There was no association between clearance of *ROS1* by C3D1 and *TP53* status (Supplementary Table 3).

**Extended Data Fig. 7:**
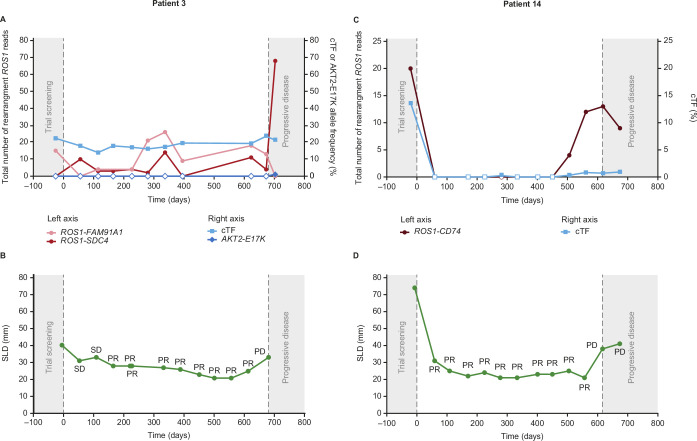
Patient cases assessing the relationship between ctDNA levels and tumor response over the duration of entrectinib treatment. Treatment started at t0. Empty data points represent when ctDNA was not detected. Patient 3 (**A**–**B**) had consistent levels of ctDNA throughout the duration of treatment (**A**) and levels of ctDNA were not associated with tumor response (**B**). Clinical presentation for Patient 3: 51-year-old male with one target lesion in the lung and one non-target lesion in the lymph node. Disease progression was due to a small increase in the target lesion in the lung; non-target lesion stayed stable. After disease progression, the patient continued treatment with entrectinib and received five subsequent lines of therapy. As of April 2023, the patient was still alive. Patient 3 had two *ROS1* fusion proteins (*ROS1-FAM91A1* and *ROS1-SDC4*), indicative of two cell populations and an *AKT2* resistance mutation was identified at treatment discontinuation. Patient 14 (**C**–**D**) responded to treatment with entrectinib cleared ctDNA by day 59 (**C**) and ctDNA levels rebounded before radiological progression (**D**). Clinical presentation for Patient 14: 37-year-old male with three target lesions (one in the lung and two in the lymph nodes) and one non-target lesion. Disease progression was due to progression in the target lesions in the lymph nodes and the presence of two new lesions in the lymph nodes. There was no disease progression in the primary target lesion (lung) and non-target lesions were stable. After disease progression, the patient continued treatment with entrectinib and received seven subsequent lines of therapy. As of October 2023, the patient was still alive. cTF, circulating tumor fraction; PR, partial response; PD, progressive disease; SD, stable disease; SLD, sum of the longest diameters.

Additional biomarker analyses were conducted to determine whether there is a relationship between changes in ctDNA levels, as measured by *ROS1* fusion levels and cTF, and tumor response over the duration of treatment (Extended Data Fig. 7). We present two case studies that followed patients from trial screening to treatment discontinuation, including multiple on-treatment samples collected at every other treatment cycle. One patient who responded to treatment with entrectinib had consistent levels of ctDNA throughout the duration of treatment (Extended Data Fig. 7a,b). Conversely, another patient who responded to treatment with entrectinib cleared ctDNA by day 59, and ctDNA levels rebounded before radiological progression (Extended Data Fig. 7c,d). These case studies demonstrate that there is no clear relationship between levels of ctDNA and clinical response.

### Molecular mechanisms of resistance to entrectinib

Molecular analysis of acquired mechanisms of resistance was conducted in plasma samples from patients that experienced disease progression during treatment with entrectinib and had samples available from the time of treatment discontinuation (*n* = 20). Most patients (*n* = 14, 70.0%) had *ROS1* fusions identified at treatment discontinuation. One patient appeared to have a different ROS1 fusion partner at screening and treatment discontinuation, but this may be due to technical differences between the assays used (Extended Data Table 1). There was no association between the identified *ROS1* fusion partner and clearance at C3D1 and *TP53* status (Extended Data Table 1). Details of other emerging mutations identified at treatment discontinuation, and their association with disease biology (known, likely and unknown), are listed in Extended Data Table 2. Thirty emerging mutations (29 unique mutations) were identified at treatment discontinuation from 12 patients; of these, two patients had a resistance-associated, *ROS1* short-variant G2032R mutation (Extended Data Table 2).

## Discussion

The BFAST trial evaluated entrectinib in treatment-naive patients with *ROS1*-positive, advanced/metastatic NSCLC identified solely by liquid biopsies. BFAST Cohort D met its primary endpoint; the confirmed ORR per investigator in this analysis was 81.5% (95% CI: 68.6–90.8) and was above the protocol-defined threshold of 70.4%, suggesting that these data are consistent with those from the historical analysis of entrectinib in patients with *ROS1*-positive NSCLC identified by tissue-based testing (investigator-assessed ORR: 73.4% (95% CI: 63.6–82.0), data cutoff May 2019, ≥12 months of follow-up, *n* = 94). Furthermore, entrectinib demonstrated durable responses and survival: median DoR was 13.0 months (95% CI: 6.3–18.4) by investigator assessment (16.7 months (95% CI: 5.6–24.0) by IRF) and median PFS was 12.9 months (95% CI: 8.7–18.5) by investigator assessment (14.8 months (95% CI: 7.2–24.0) by IRF). OS data were immature but the 12-month OS rate was high, at 79%.

The clinical benefit of entrectinib demonstrated in BFAST Cohort D is consistent with that previously reported from the integrated analysis of three phase 1/2 studies of entrectinib in patients who were selected using tissue-based testing methods^23–26^. In the integrated analysis, as of 1 May 2019, 94 patients were enrolled with ≥12 months of follow-up and the median duration of follow-up was comparable to that from BFAST (20.9 versus 18.3 months in BFAST). At this data cutoff, median DoR was 16.4 months (95% CI: 13.1–18.5) and median PFS was 14.5 months (95% CI: 10.0–17.4) (all by investigator, data unpublished). OS data were immature, with only 27% of events recorded, and the 12-month OS rate was 83% (95% CI: 0.8–0.9; data unpublished). BFAST Cohort D was designed to demonstrate consistency with the integrated analysis of entrectinib in terms of investigator-assessed ORR, and the primary endpoint was met. Limited conclusions can be drawn on the observed numerical differences in DoR and PFS between the two datasets, which may be due to differences between the trial populations, such as using liquid biopsies for patient selection that required detectable ctDNA at baseline, which has been shown to be positively correlated with higher tumor burden^11,31,32^, or other potentially prognostic factors, such as the prevalence of *TP53* comutations^11,33,34^. Despite these potential differences, it is important to note that the integrated analysis of entrectinib remains the most relevant dataset for comparison of the results of BFAST Cohort D, because it is the only other analysis of patients with *ROS1*-positive, advanced/metastatic NSCLC who have been treated with entrectinib.

Other ROS1 inhibitors are also approved and/or in development for the treatment of *ROS1-*positive, advanced/metastatic NSCLC. Crizotinib is approved for the treatment of *ROS1-*positive, advanced NSCLC (investigator-assessed ORR 72% (95% CI: 58–84))^35^; lorlatinib, taletrectinib and repotrectinib are next-generation ROS1 inhibitors that are currently under investigation for the treatment of patients with *ROS1*-positive NSCLC who are treatment-naive and who have received previous treatment, including ROS1 TKIs^36–38^. Next-generation ROS1 inhibitors are in the early stages of clinical development and have demonstrated promising antitumor activity^36–38^. It is important to note that, in the clinical studies of ROS1 inhibitors, patients were identified by tissue-based biomarker testing and there are inherent differences between study populations that make cross-trial comparisons with BFAST inappropriate.

Brain metastases occur in approximately 40% of patients with *ROS1-*positive, advanced NSCLC, and there is a need for CNS-active treatments for these patients^19–21^. Entrectinib was specifically designed to penetrate the blood–brain barrier and has demonstrated activity within the CNS^22,23,39–42^. In BFAST Cohort D only four patients had baseline CNS metastases by investigator, and two of these achieved a partial response. Due to the low incidence of CNS disease in this cohort, intracranial efficacy could not be assessed. Time to CNS progression was assessed in all patients: the 12-month CNS progression-free rate was 86.4% by IRF and the median was not reached. These results suggest a role for entrectinib in delaying or preventing the development of CNS metastases, even in patients without baseline CNS disease. However, further data are required to make any definitive conclusions. It is important to note that CNS follow-up was mandated only for patients with baseline CNS metastases.

The intracranial benefit of entrectinib has been previously reported in the integrated analysis of patients with *ROS1*-positive, advanced NSCLC^23–26^. However, the CNS efficacy of crizotinib is not well defined; in a phase 1 trial of crizotinib in patients with *ROS1-*positive, advanced NSCLC, patients with CNS metastases were excluded^43^. Preliminary results from the next-generation ROS1 inhibitors repotrectinib and taletrectinib suggest that they have activity in the CNS^36–38^. Evidence remains limited for the CNS efficacy of lorlatinib^44^. As such, there is an unmet need to understand the comparative efficacy of TKIs, especially in the CNS. A head-to-head, randomized, open-label, phase 3 trial of entrectinib versus crizotinib in patients with *ROS1*-positive NSCLC (NCT04603807) is currently ongoing, and will assess both systemic and intracranial endpoints^45^.

Overall, the safety profile of entrectinib in BFAST was generally consistent with that reported previously^23–26^. Entrectinib was well tolerated and no new safety signals were identified. The high median dose intensity (>97%) indicates that almost all patients received the full, planned dose and that dose reductions and/or interruptions did not impact overall dose exposure.

The prevalence of *ROS1* fusions identified in this study was 1.8%, which is consistent with that previously reported in studies using tissue-based testing (1–2%)^15,17,18,46^, providing further evidence that liquid biopsies are an appropriate methodology for identification of patients who may benefit from entrectinib treatment^11^. Patients enrolled in BFAST may have undergone previous tissue-based testing and been preselected for screening in BFAST, which may have enriched the reported prevalence.

Post hoc exploratory analyses were conducted to further characterize the patient population and identify any potential prognostic biomarkers; however, because the number of patients in all biomarker analyses was low, the results should be interpreted with caution. Clearance of ctDNA from baseline to C3D1 may be prognostic of clinical outcomes, because patients who had cleared *ROS1* by C3D1 had prolonged survival outcomes compared with those who had not. These results are in line with findings from other studies that have also shown clearance of ctDNA from baseline to C3 to be associated with improved clinical outcomes^47^.

The presence of m*TP53*, the most common comutation found in these patients, was associated with worse prognosis, which is consistent with previous reports^11,33,34^. However, it should be noted that m*TP53* was more prevalent in BFAST Cohort D than previously reported^11,34^. A potential reason for this difference may be due to *TP53* being frequently mutated in clonal hematopoiesis of indeterminate potential (CHIP)^48^; in BFAST it was not possible to determine the contribution of CHIP to the prevalence of m*TP53* (ref. ^11^). Alternatively, m*TP53* might have been more prevalent in BFAST due to the selection of patients with ctDNA, which may have enriched the prevalence of m*TP53*.

*CD74* and *EZR* have previously been reported as the most common fusion partners for *ROS1* (refs. ^23,24,49,50^), consistent with the findings in BFAST Cohort D. In regard to *CD74* there is contradictory evidence as to whether the presence of this fusion partner is prognostic of survival outcomes in patients treated with entrectinib or crizotinib^24,49,50^. In our analysis, clinical outcomes were comparable between patients with *CD74-ROS1* fusions and those with other *ROS1* fusion partners, suggesting that *CD74* may not be a prognostic factor in this group of patients. Previous studies have suggested that patients with *EZR-ROS1* fusions have better clinical outcomes compared with patients with other *ROS1* fusions treated with entrectinib or crizotinib^24,48^. However, in our analysis, clinical outcomes were comparable between patients with *EZR-ROS1* fusions and those with other *ROS1* fusion partners, suggesting that *EZR* may not be a prognostic factor in this group of patients.

Post hoc exploratory analyses were conducted to determine whether the level of ctDNA in the blood had any prognostic value in these patients, but did not find an association between cTF levels (an estimate of tumor fraction) and clinical outcomes. These findings contrast with a real-world study showing that higher levels of ctDNA are associated with poorer prognosis across four advanced types of cancer (prostate, breast, NSCLC and colorectal); however, patients in that study were identified using tissue-based testing and included some who were ctDNA negative^51^. In BFAST Cohort D, cTF levels did correlate with tumor burden as measured by SLD. These results are consistent with data from the IMpower150 study of first-line atezolizumab in combination with chemotherapy and/or bevacizumab for patients with advanced NSCLC, and further support the hypothesis that higher levels of ctDNA may not be associated with poorer prognosis in all cases^47^. However, it is important to note that patients with metastatic disease can have widely disseminated disease and/or multiple nontarget lesions that are not considered with the response evaluation criteria in solid tumors (RECIST) v.1.1 criteria, and therefore SLD may not accurately reflect actual tumor burden^52^. Due to the small number of patients in BFAST, further research is required to validate the findings from these exploratory biomarker analyses. Furthermore, because BFAST is a single-arm study, it is not possible to deduce whether any of these factors are predictive biomarkers of benefit from entrectinib.

Lastly, preliminary analyses were conducted to determine whether there is a relationship between levels of ctDNA and clinical response; no clear relationship was identified in the case studies presented. Additional studies are needed to establish whether ctDNA re-emergence precedes radiographic progression and whether continuous ctDNA testing may be a useful tool to inform treatment decisions.

Potential molecular mechanisms of resistance to entrectinib were explored in patients who had disease progression and available plasma samples from the time of treatment discontinuation. Potential acquired-resistance mutations were identified, including *ROS1* G2032R, which has previously been associated with resistance to lorlatinib and entrectinib^11,53^. Additional analyses are required to fully elucidate the mechanisms of resistance in this study.

Limitations of the present study include the small sample size and lack of a comparator arm. In addition, this analysis took place after a relatively short follow-up time (the last patient enrolled was followed up for ~13 months, median duration of follow-up was 18.3 months) and 15 patients (27%) were still being treated at data cutoff. Further follow-up is needed to accurately assess survival in these patients. Another potential limitation of this study is that two different clinical trial assays, FoundationOneLiquid CDx and FoundationACT, were used to assess clinical samples, which may have introduced variability. However, because additional testing demonstrated high concordance between the two assays (positive predictive agreement 96.9%), we expect this variability to be minimal for the genes covered by both assays. A limitation of liquid biopsies is that the use of a test based on ctDNA depends on the tumor shedding into the blood, and therefore some patients (for example, those with a low tumor burden and less shedding) may not be assessable by this method^13,54^. However, liquid biopsies may improve biopsy turnaround times (typical turnaround time of the FoundationOneLiquid CDx clinical trial assay is ≤10 days from receipt of specimen) and increase access to targeted therapies for patients who are unable to receive a tissue-based biopsy or who have insufficient tissue on which to perform biomarker analyses^55^.

In conclusion, these data support the clinical applicability of liquid biopsies to inform clinical decisions, and provide further evidence that entrectinib is effective and well tolerated in patients with *ROS1*-positive, advanced/metastatic NSCLC.

## Methods

### Study design and patients

BFAST (NCT03178552) is a global, open-label, multicohort study (Fig. 1). The study protocol is available in Supplementary Data. Eligible patients were ≥18 years of age; had previously untreated, unresectable, advanced or metastatic (stage IIIB or IV) NSCLC that was not amenable to concomitant chemoradiation; had Eastern Cooperative Oncology Group performance status (ECOG PS) of 0–2; had life expectancy ≥12 weeks; and had measurable disease by RECIST v.1.1. Patients who had received previous treatment for nonmetastatic disease (neoadjuvant or adjuvant chemotherapy, radiotherapy or chemoradiotherapy) must have been treatment free for ≥6 months before enrollment in the study. Patients with brain metastases at screening were eligible if asymptomatic and/or previously treated, and patients who had received brain radiotherapy must have completed treatment ≥14 days before the start of entrectinib treatment. Sex was not considered in the study design, and both females and males were enrolled in the study. Sex was self-reported and information regarding gender was not collected.

Patients were screened prospectively for actionable mutations using the blood-based NGS assays FoundationOne®Liquid CDx clinical trial assay or Foundation Medicine Assay for Circulating Tumor DNA (FoundationACT™; Foundation Medicine, Inc., Cambridge, MA); details of these assays have been described previously^13^. *ROS1* rearrangements were defined as fusions between *ROS1* and a known partner gene regardless of frame, or in-frame fusions between *ROS1* and a novel partner gene. In addition, the *ROS1* breakpoint must have occurred before the start of the kinase domain. Patients identified as having *ROS1*-positive NSCLC and who met the cohort-specific eligibility criteria were enrolled into Cohort D of BFAST. Enrollment was based solely on liquid biopsy results and was established irrespective of locally assessed, tissue-based results. Although tissue collection and central testing of tissue to determine *ROS1* status were not required, tissue availability for molecular testing and local biomarker test results could be reported by the investigator.

The study was performed in accordance with the principles of the Declaration of Helsinki, and all patients provided written informed consent for initial blood screening and enrollment into a treatment cohort. Protocols were approved by the relevant institutional review boards at each study site. The study protocol was approved by the institutional review boards of participating institutions, including the Ontario Cancer Research Ethics Board (Princess Margaret Cancer Center, William Osler Health System Brampton Civic Hospital and Sunnybrook Health Sciences Center) and the University of Saskatchewan Biomedical Research Ethics Board (Saskatoon Cancer Centre).

### Treatment and assessments

Patients with *ROS1*-positive NSCLC received entrectinib at 600 mg orally once per day until either disease progression (according to RECIST v.1.1), unacceptable toxicity, withdrawal of consent, study termination by sponsor or death (which ever occurred first). Tumor assessments were performed at baseline and every 8 weeks thereafter. Dose reductions in increments of 200 mg were allowed for adverse events, and entrectinib treatment could also be interrupted for a maximum of 28 days. Brain imaging (computerized tomography scan allowed if magnetic resonance imaging was not feasible) was not mandated beyond baseline in patients without baseline CNS disease.

### Study endpoints

The primary endpoint was confirmed ORR according to investigator assessment, defined as the proportion of patients with CR or PR according to RECIST v.1.1. Confirmation of response was required and determined by two separate tumor assessments ≥4 weeks apart. Secondary endpoints were CBR, DoR and PFS by investigator assessment (according to RECIST v.1.1); ORR, CBR, DoR and PFS by IRF assessment (according to RECIST v.1.1); OS; time to CNS progression (by investigator and IRF assessment according to RECIST v.1.1); and safety. Secondary endpoint definitions are as follows: CBR is the proportion of patients with CR, PR or SD maintained for ≥24 weeks; DoR is the time from confirmed CR/PR to occurrence of a progression event or death; PFS is the time from first treatment to documentation of disease progression or death, whichever occurred first; OS is the time from first treatment to date of death by any cause; and time to CNS progression is the time from first treatment to radiographic evidence of CNS progression (defined as the development of new CNS lesions and/or progression of pre-existing baseline CNS lesions). Investigator-assessed ORR in patients with CNS metastases at baseline was an exploratory endpoint. All biomarker analyses were post hoc exploratory. The incidence and severity of AEs were graded according to the National Cancer Institute Common Terminology Criteria for Adverse Events v.4.0.

### Statistical analysis

Determination of sample size was based on demonstration of data consistency between BFAST (blood-selected patients) and the integrated analysis of three clinical trials of entrectinib (tissue-selected patients). Assuming that the established ORR seen with entrectinib in the integrated analysis was 75% (the integrated analysis was ongoing at the time BFAST Cohort D was initiated), BFAST planned to enroll 50 patients to provide a 75% chance that the lower limit of the two-sided 95% CI (using the Clopper–Pearson method) around the point estimate of ORR in patients selected by liquid biopsy would be >72% (thus preserving at least 75% of the ORR observed with entrectinib in the integrated analysis in which patients were selected using tissue-based testing). The protocol prespecified preservation of 75% ORR to allow for potential differences between an entirely ctDNA-positive population versus a historical control, and in line with the approach taken in other single-arm cohorts in the BFAST study. With the actual number of enrolled and measurable patients (*n* = 54), an ORR of ≥70.4% (95% CI: 56–82%) (*n* = 37 responders) was required for Cohort D to meet its primary endpoint.

Kaplan–Meier methodology was used to estimate median DoR, PFS and OS with corresponding 95% CIs. Concordance between assays used to identify *ROS1* fusions (FoundationACT and FoundationOneLiquid CDx clinical trial assay) was calculated as positive or negative percentage agreement and has been described previously^13^. Clinical analyses were performed using SAS (v.9.04) and post hoc exploratory analyses were performed in R (v.3.5.2). Statistics for all post hoc exploratory analyses are descriptive.

### Post hoc exploratory biomarker analyses

Circulating tumor fraction is an estimate of the amount of ctDNA in a sample and is associated with high sensitivity for the detection of actionable alterations^56^. Two complementary approaches were used to measure cTF: the proprietary tumor fraction estimator (TFE) and maximum somatic allele frequency (MSAF). TFE relies on tumor aneuploidy information to calculate deviations in sequencing coverage, which is most reliable when the tumor fraction is >10%. In the absence of robust tumor aneuploidy information, MSAF is used to estimate cTF^57^. MSAF is based on the allele frequency of short variants (SNV/indels) alone, and not on any fusions detected; if only a fusion is detected without any short variants, MSAF will be 0%. Hazard ratios (HRs) and 95% CIs for clinical outcomes (DoR and PFS) were estimated using Cox regression models stratified at specified cTF thresholds. The correlation between baseline cTF and tumor size (measured by SLD) was estimated using Pearson’s correlation coefficient with 95% CI.

Patients were included in the *ROS1* clearance analysis if they had plasma samples available from C3D1 and were evaluated for *ROS1* ctDNA. *ROS1* clearance at C3D1 was defined as no detectable *ROS1* alterations at this timepoint.

Molecular analysis of resistance mutations was conducted in plasma samples from patients that experienced disease progression during treatment with entrectinib and had samples available from the time of treatment discontinuation. Emerging mutations were defined as mutations that were not detected at baseline but were detected at treatment discontinuation. For patients whose samples were analyzed by FoundationOneLiquid CDx clinical trial assay at baseline and treatment discontinuation, all 311 genes assayed by that assay were included in the analysis of emerging mutations. For patients whose samples were analyzed by FoundationACT at baseline and treatment discontinuation, or by FoundationACT at baseline and FoundationOneLiquid CDx clinical trial assay at treatment discontinuation, analysis of emerging mutations was restricted to the 62 genes captured by both FoundationACT and FoundationOneLiquid CDx clinical trial assay (*ROS1* is evaluated in both assays).

### Reporting summary

Further information on research design is available in the Nature Portfolio Reporting Summary linked to this article.

## Online content

Any methods, additional references, Nature Portfolio reporting summaries, source data, extended data, supplementary information, acknowledgements, peer review information; details of author contributions and competing interests; and statements of data and code availability are available at 10.1038/s41591-024-03008-4.

### Supplementary information

Supplementary Information Supplementary Data: concordance between assays used to detect *ROS1* fusions, and Tables 1–3 and Fig. 1.

Reporting Summary

## Figures and Tables

**Extended Data Table 1:**
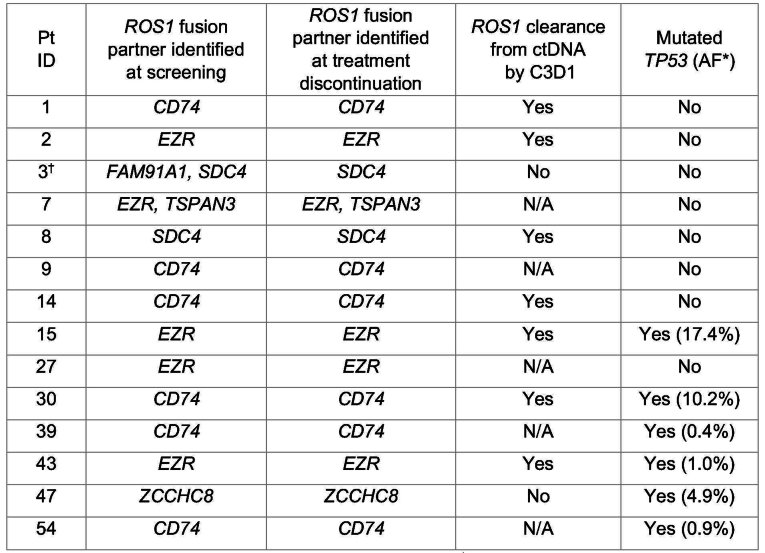
*ROS1* fusion partners identified at screening and at treatment discontinuation, status of *ROS1* clearance from ctDNA by C3D1, and TP53 status, by patient

*Maximum AF of *TP53* alterations detected at baseline; ^†^Different fusion partners identified at screening (FoundationACT™) and treatment discontinuation (F1LiqCDx) may be due to technical differences between the different assays used.

*ROS1* clearance from ctDNA by C3D1 could not be assessed in five patients due to lack of sample; denoted in the table as N/A.

AF, allele frequency; C3D1, cycle 3 day 1; ctDNA, circulating tumor DNA; N/A, not applicable; *ROS1*, ROS proto-oncogene 1.

**Extended Data Table 2:**
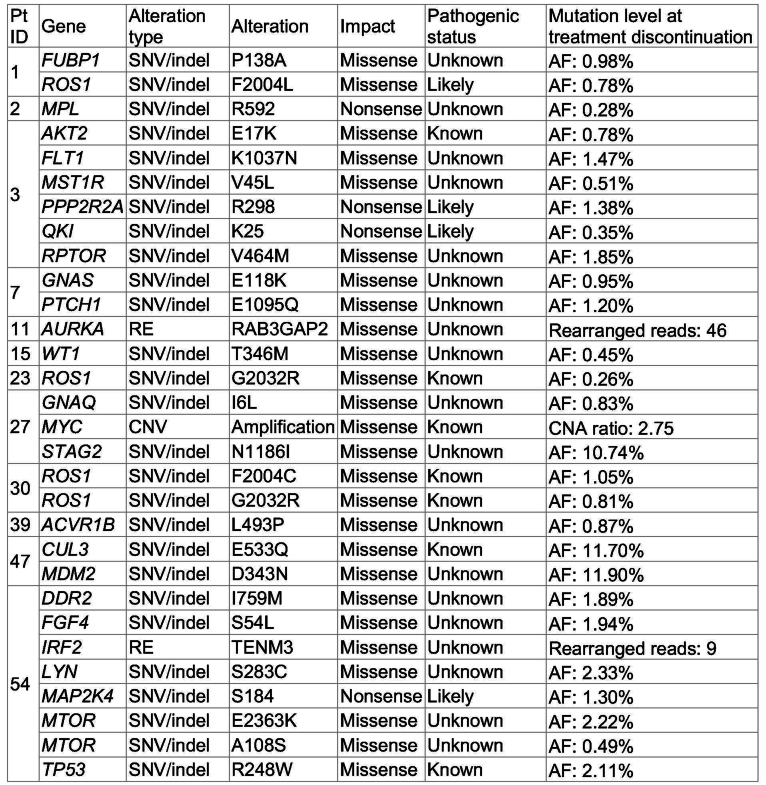
Emerging mutations identified in patients at treatment discontinuation

AF, allele frequency; CNA, copy number alteration, CNV, copy number variant; RE, rearrangement; SNV/indel, short variant.

## Data Availability

All clinical and ctDNA data for BFAST Cohort D are deposited to the European Genome-Phenome Archive under accession no. EGAS50000000105. For up-to-date details on Roche’s Global Policy on the Sharing of Clinical Information and how to request access to related clinical study documents, see https://go.roche.com/data_sharing. Anonymized records for individual patients across more than one data source external to Roche cannot, and should not, be linked because of a potential increase in the risk of patient reidentification.
