# Supplementary material for: Entrectinib in ROS1-positive advanced non-small cell lung cancer: the phase 2/3 BFAST trial
Source: Nat Med. 2024 Jun 19;30(7):1923–32. doi: 10.1038/s41591-024-03008-4 (PMC11271410; doi:10.1038/s41591-024-03008-4)
Supplement: Supplementary file 1 — Supplementary Data: concordance between assays used to detect ROS1 fusions, and Tables 1–3 and Fig. 1. [file 41591_2024_3008_MOESM1_ESM.pdf]

nature medicine

Supplementary information https://doi.org/10.1038/s41591-024-03008-4

Entrectinib in *ROS1*-positive advanced non-small cell lung cancer: the phase 2/3 BFAST trial

In the format provided by the authors and unedited

Supplementary materials

Supplementary Data│Concordance between assays used to detect *ROS1* fusions

Of the 33 patients enrolled by FoundationACT™, 32 had retrospective testing by FoundationOne®Liquid CDx clinical trial assay, which passed quality control checks including ctDNA fraction (cTF) >0, and had no detected contamination. The Positive Predictive Agreement between FoundationACT™ and FoundationOne®Liquid CDx clinical trial assay was 96.9% (31/32). The one patient that did not have a *ROS1* fusion detected in both samples had very low cTF in the F1CDx C1D1 sample (0.21%).

Supplementary Table 1 │Confirmed best overall response by investigator, by *ROS1* fusion partner

ROS1 fusion partner*	CR/PR, % (n)	SD/PD, % (n)
CD74 (n = 30)	80.0 (24)	20.0 (6)
Other (n = 24)	83.3 (20)	16.7 (4)

*In patients with baseline measurable disease (*N* = 54).
CR, complete response; PR, partial response; PD, progressive disease;
ROS1, ROS proto-oncogene 1; SD, stable disease.

Supplementary Table 2 │Confirmed best overall response by investigator, in patients who cleared *ROS1* at C3D1 and those who did not

cBOR evaluable patients with plasma samples from C3D1 (N = 35)	*ROS1* cleared at C3D1, % (n)	*ROS1* not cleared at C3D1, % (n)
Non-responder (n = 6)	66.7 (4)	33.3 (2)
Responder (n = 29)	89.7 (26)	10.3 (3)

36 patients had plasma samples from C3D1, one of these patients had non-measurable disease and was not included in this analysis of responders / non-responders.
C3D1, cycle 3 day 1; cBOR, confirmed best overall response;
ROS1, ROS proto-oncogene 1.

Supplementary Table 3 │ Rates of *ROS1* clearance by C3D1 in patients with and without TP53 mutations at baseline

Patients assessed for *ROS1* clearance at C3D1 (n=36)	*ROS1* cleared at C3D1, % (n)	*ROS1* not cleared at C3D1, % (n)
No *TP53* mutation at baseline	87.0 (20)	13.0 (3)
TP53 mutation at baseline	84.6 (11)	15.4 (2)

C3D1, cycle 3 day 1; *ROS1,* ROS proto-oncogene 1.

Supplementary Fig. 1 │PFS by investigator with different cTF thresholds

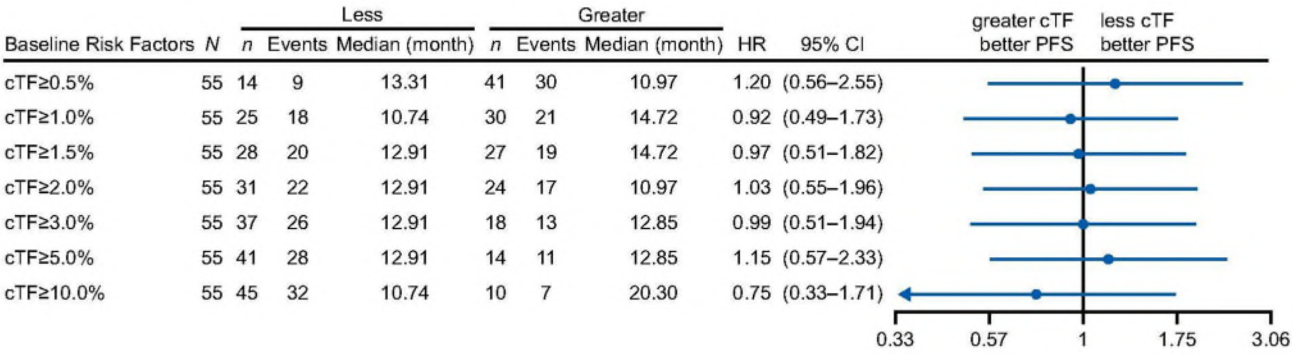

CI, confidence interval; cTF, ctDNA fraction; PFS, progression-free survival.
