# Supplementary material for: Entrectinib in ROS1-positive advanced non-small cell lung cancer: the phase 2/3 BFAST trial
Source: Nat Med. 2024 Jun 19;30(7):1923–32. doi: 10.1038/s41591-024-03008-4 (PMC11271410; doi:10.1038/s41591-024-03008-4)
Supplement: Supplementary file 2 — Reporting Summary [file 41591_2024_3008_MOESM2_ESM.pdf]

nature portfolio

Corresponding author(s): Solange Peters

Last updated by author(s): Apr 10, 2024

Reporting Summary

Nature Portfolio wishes to improve the reproducibility of the work that we publish. This form provides structure for consistency and transparency in reporting. For further information on Nature Portfolio policies, see our Editorial Policies and the Editorial Policy Checklist.

Statistics

For all statistical analyses, confirm that the following items are present in the figure legend, table legend, main text, or Methods section.

n/a	Confirmed	
☐	☒	The exact sample size (*n*) for each experimental group/condition, given as a discrete number and unit of measurement
☐	☒	A statement on whether measurements were taken from distinct samples or whether the same sample was measured repeatedly
☐	☒	The statistical test(s) used AND whether they are one- or two-sided<br>*Only common tests should be described solely by name; describe more complex techniques in the Methods section.*
☐	☒	A description of all covariates tested
☐	☒	A description of any assumptions or corrections, such as tests of normality and adjustment for multiple comparisons
☐	☒	A full description of the statistical parameters including central tendency (e.g. means) or other basic estimates (e.g. regression coefficient) AND variation (e.g. standard deviation) or associated estimates of uncertainty (e.g. confidence intervals)
☐	☒	For null hypothesis testing, the test statistic (e.g. *F*, *t*, *r*) with confidence intervals, effect sizes, degrees of freedom and *P* value noted<br>*Give P values as exact values whenever suitable.*
☒	☐	For Bayesian analysis, information on the choice of priors and Markov chain Monte Carlo settings
☒	☐	For hierarchical and complex designs, identification of the appropriate level for tests and full reporting of outcomes
☐	☒	Estimates of effect sizes (e.g. Cohen's *d*, Pearson's *r*), indicating how they were calculated

Our web collection on statistics for biologists contains articles on many of the points above.

Software and code

Policy information about availability of computer code

Data collection	No software was used for the collection of data
Data analysis	Exploratory analyses were performed in R (v3.5.2). Clinical analyses were performed using SAS (v9.04)

For manuscripts utilizing custom algorithms or software that are central to the research but not yet described in published literature, software must be made available to editors and reviewers. We strongly encourage code deposition in a community repository (e.g. GitHub). See the Nature Portfolio guidelines for submitting code & software for further information.

Data

Policy information about availability of data

All manuscripts must include a data availability statement. This statement should provide the following information, where applicable:

- Accession codes, unique identifiers, or web links for publicly available datasets
- A description of any restrictions on data availability
- For clinical datasets or third party data, please ensure that the statement adheres to our policy

Data availability statement that is provided in the manuscript:
All clinical and ctDNA data for BFAST cohort D are deposited to the European Genome Phenome Archive under accession number EGAS50000000105. For up-to-date details on Roche's Global Policy on the Sharing of Clinical Information and how to request access to related clinical study documents, see here: https://go.roche.com/data_sharing. Anonymized records for individual patients across more than one data source external to Roche cannot, and should not, be linked

because of a potential increase in the risk of patient re-identification.

Research involving human participants, their data, or biological material

Policy information about studies with human participants or human data. See also policy information about sex, gender (identity/presentation), and sexual orientation and race, ethnicity and racism.

Reporting on sex and gender	We can confirm that the findings of this research does not apply to only one sex or gender and therefore this is not stated in the title or abstract.<br>Sex and/or gender was not considered in the study design, and we confirm that the sex of participants was self-reported/assigned by the investigator.<br>Disaggregated data for sex has been reported in Table 1 (32 patients were female and 23 patients were male) and we confirm that consent has been obtained for reporting and sharing individual-level data.<br>No differences in outcomes have previously been reported between males and females treated with entrectinib, who were selected using tissue-based testing. In light of this, sex-based analyses in a patient population selected for treatment with entrectinib based on blood-based data are unlikely to yield different results.<br>Gender was not reported in this study as it was not relevant.
Reporting on race, ethnicity, or other socially relevant groupings	Self-reported race of patients has been reported in Table 1.
Population characteristics	Eligible patients were >18 years old, had previously untreated, unresectable, advanced or metastatic (stage IIIB or IV) NSCLC that was not amenable to concomitant chemoradiation; had an ECOG PS of 0–2; had life expectancy >12 weeks; and had measurable disease RECIST v1.1. Patients indentifed to have ROS1-positive NSCLC by blood-based NGS assays (FoundationOne®Liquid CDx or FoundationACT™) were eligible for Cohort D<br>Baseline characteristics reported in Table 1 are generally well balanced
Recruitment	Patients were enrolled at 120 centers across 25 countries. These sites were selected based on factors such as patients population availability and site staff expertise in lung cancer and the conduct of clinical trials.<br>Patients were recruited to be screened and enrolled at the discretion of the primary investigator. All patients had a central blood-based NGS screening that informed investigators as to whether their patient was eligible for a treatment cohort. Investigators were responsible for assessing a patients eligibility to participate in a treatment cohort. Our protocol design allowed for the screening of a representative sample of patients with advanced NSCLC<br><br>Patients enrolled in BFAST may have undergone prior tissue-based testing and been pre-selected for screening in BFAST which may have enriched the reported prevalence of ROS1 fusions in this population.
Ethics oversight	The study was performed in accordance with the principles of the Declaration of Helsinki and all patients provided written informed consent for initial blood screening and enrollment into a treatment cohort. The study protocol was approved by institutional review boards of participating institutions, including the Ontario Cancer Research Ethics Board (OCREB) (Princess Margaret Cancer Center, William Osler Health System Brampton Civic Hospital, and Sunnybrook Health Sciences Center) and the University of Saskatchewan Biomedical Research Ethics Board (Saskatoon Cancer Centre).

Note that full information on the approval of the study protocol must also be provided in the manuscript.

Field-specific reporting

Please select the one below that is the best fit for your research. If you are not sure, read the appropriate sections before making your selection.

☒ Life sciences ☐ Behavioural & social sciences ☐ Ecological, evolutionary & environmental sciences

For a reference copy of the document with all sections, see nature.com/documents/nr-reporting-summary-flat.pdf

Life sciences study design

All studies must disclose on these points even when the disclosure is negative.

Sample size	Determination of sample size was based on demonstration of data consistency between BFAST (blood-selected patients) and the integrated analysis of three clinical trials of entrectinib (tissue-selected patients). Assuming the established ORR seen with entrectinib in the integrated analysis was 75% (the integrated analysis was ongoing at the time BFAST Cohort D was initiated), BFAST planned to enroll 50 patients to provide a 75% chance that the lower limit of the two-sided 95% CI (using Clopper-Pearson method) around the point estimate of ORR in patients selected by liquid biopsy would be >72% (thus preserving at least 75% of the ORR observed with entrectinib in the integrated analysis in which patients were selected using tissue-based testing). The protocol pre-specified preserving 75% ORR to allow for potential differences between an entirely ctDNA-positive population versus a historical control, and in line with the approach taken in other single-arm cohorts in the BFAST study.<br><br>Between 11 January 2018 and 9 December 2020, 5,220 patients were screened, and 92 patients were identified to have ROS1-positive, advanced/metastatic NSCLC by liquid biopsies. Of these 92 patients, 55 treatment naïve patients were enrolled, and 54 of them had measurable disease (Table 1). The last patient included in this analysis was enrolled on 1 October 2020, and the data cut-off was 26 November 2021.

Data exclusions	No data were excluded
Replication	No experiments were repeated or replicated
Randomization	Patients were assigned to a treatment based on the results of the blood-based screening assay
Blinding	Current study is an open label study and no blinding was required

Reporting for specific materials, systems and methods

We require information from authors about some types of materials, experimental systems and methods used in many studies. Here, indicate whether each material, system or method listed is relevant to your study. If you are not sure if a list item applies to your research, read the appropriate section before selecting a response.

Materials & experimental systems

n/a	Involved in the study	
☒	☐	Antibodies
☒	☐	Eukaryotic cell lines
☒	☐	Palaeontology and archaeology
☒	☐	Animals and other organisms
☐	☒	Clinical data
☒	☐	Dual use research of concern
☒	☐	Plants

Methods

n/a	Involved in the study	
☒	☐	ChIP-seq
☒	☐	Flow cytometry
☒	☐	MRI-based neuroimaging

Clinical data

Policy information about clinical studies

All manuscripts should comply with the ICMJE guidelines for publication of clinical research and a completed CONSORT checklist must be included with all submissions.

Clinical trial registration	NCT03178552
Study protocol	A redacted version of this protocol will be made available alongside this article
Data collection	Between 11 January 2018 and 9 December 2020, 5,220 patients were screened by blood-based NGS in the BFAST study. The last patient included in this analysis was enrolled on 1 October 2020, and the data cut-off was 26 November 2021.<br>Patients were enrolled from 120 centers across 25 countries. Academic and community oncology practices participated in the study and these sites were selected based on factors such as patients population availability and site staff expertise in lung cancer and the conduct of clinical trials.
Outcomes	The primary endpoint was confirmed objective response rate (ORR) per investigator (INV).<br>Secondary endpoints were clinical benefit rate (CBR) , duration of response (DoR), and progression-free survivavl (PFS) by INV; ORR, CBR, DoR, PFS by independent review facility (IRF); overall survival; time to CNS progression (by INV and IRF); and safety.<br>All biomarker analyses are exploratory endpoints and were not pre-specfied in the protocol.

Plants

Seed stocks	n/a
Novel plant genotypes	n/a
Authentication	n/a
